# Stereotactic Re-irradiation for Local Recurrence in the Prostatic Bed After Prostatectomy: Preliminary Results

**DOI:** 10.3389/fonc.2019.00071

**Published:** 2019-02-15

**Authors:** Jonathan Olivier, Laurent Basson, Philippe Puech, Thomas Lacornerie, Arnauld Villers, Jennifer Wallet, Eric Lartigau, David Pasquier

**Affiliations:** ^1^Academic Department of Radiation Oncology, Centre Oscar Lambret, Lille, France; ^2^Department of Urology, CHU Lille, Lille, France; ^3^Department of Radiology, CHU Lille, Lille, France; ^4^Department of Medical Physics, Centre Oscar Lambret, Lille, France; ^5^Department of Biostatistics, Centre Oscar Lambret, Lille, France; ^6^CRIStAL UMR CNRS 1189, Lille University, Villeneuve-d'Ascq, France

**Keywords:** prostate, cancer, salvage radiotherapy, re-irradiation, prostatic bed, recurrence, stereotactic radiation body therapy

## Abstract

**Objectives:** To report the preliminary results of salvage re-irradiation in the prostatic bed after radical prostatectomy and salvage external beam radiation therapy (EBRT) using robotic stereotactic body radiation therapy (SBRT) with Cyberknife® for local recurrence of prostate cancer.

**Materials and Methods:** Retrospective monocentric analysis was performed on patients treated with SBRT for isolated macroscopic recurrence in the prostatic bed. All patients had radical prostatectomy and salvage or adjuvant EBRT. Local recurrence was documented using magnetic resonance imaging (MRI) and positron emission tomography (PET). Biochemical recurrence was defined as 2 rises in prostate-specific antigen (PSA) of ≥ 0.2 ng/mL above nadir. Internal gold fiducials were used for the tracking of tumor motion during SBRT. The prescription dose was 36 Gy in 6 fractions for all patients. Toxicity was scored according to the CTCAE v4.0.

**Results:** Between July 2011 and November 2017, 12 patients were treated with SBRT for prostatic bed recurrence with a median follow-up of 34.2 (range, 3.5–64.4) months. Isolated non-metastatic recurrence in the prostatic bed was seen at MRI and PET imaging. Two patients were treated with 6 months androgen deprivation therapy (ADT) concomitant with re-irradiation. The median planning target volume was 4.5 cm^3^ (range, 1.2–13.3). A PSA decrease after SBRT was found in 10 (83%) patients. The 1 and 2 years biochemical recurrence-free survival rates were 79 and 56%, respectively. Biochemical recurrence was observed for 6 patients (50%) after a median time of 18 (4-42) months. Toxicity showed: 3 patients (25%) with grade 1 cystitis and 1 patient (8%) with acute grade 2 proctitis at 4 months. One patient (13%) had grade 1 cystitis at 12 months.

**Conclusion:** Re-irradiation for local recurrence in the prostatic bed using Cyberknife® after surgery and salvage or adjuvant EBRT is well-tolerated and associated with 2 years biochemical recurrence-free survival rates of 56%. Longer follow-up and larger series are necessary.

## Introduction

Prostate cancer (PCa) is the most common cancer in men in western countries and radical prostatectomy (RP) remains one of the standard-of-care treatment options for localized cancers (1). Despite new surgical approaches using robotics to optimize outcomes, the rate of biochemical recurrence (BCR), defined by a serum prostate-specific antigen (PSA) level >0.2 ng/mL, after primary RP remains around 20–30% (2, 3).

When PSA level increases after RP, investigations are needed to differentiate local recurrences from distant metastases. More than 50% of these recurrences are local and the most common option for local salvage therapy after RP is radiation therapy (4). External beam radiation therapy (EBRT) after RP can offer favorable PSA responses, especially when given early (PSA < 0.6 ng/mL). Unfortunately, 45–65% of men treated using salvage radiation therapy after RP will experience a second BCR at 5 years (5).

There are no guidelines regarding the management of increasing PSA level after RP and salvage RT (6). Historically, local failure after salvage radiation is often managed with androgen deprivation therapy (ADT) to slow disease progression (7); however, ADT is linked with a poor quality of life (8), and some patients may be suitable for another local salvage treatment. The development of prostate magnetic resonance imaging (MRI) allows the identification and localization of local recurrence with higher precision (9). MRI-guided local therapies for new recurrent PCa could delay or avoid the use of systemic therapies.

Stereotactic body radiation therapy (SBRT) is an interesting locoregional treatment option for limited sites of recurrence. SBRT as primary PCa treatment has shown excellent local control with limited toxicities (10–12). There are several active clinical protocols comparing this technique to conventional treatments. To date, around 30 patients treated using SBRT for local recurrence after RP and salvage EBRT have been described in the literature (13–15). We report our preliminary results of salvage re-irradiation SBRT using Cyberknife® (Accuray Incorporated, Sunnyvale, California) in the prostatic bed for local recurrences of PCa.

## Materials and Methods

### Patients

Between July 2011 and October 2017, 12 consecutive patients were treated with SBRT for a local recurrence after RP and salvage or adjuvant EBRT using the CyberKnife® System at the Oscar Lambret Comprehensive Cancer Center (Lille, France) as decided in a multidisciplinary meeting. Data were retrospectively collected. The study complies with the “reference methodology” adopted by the French Data Protection Authority (CNIL) and patients did not object to the use of their clinical data for the research purpose. As retrospective study, ethics committee approval was not required per the local legislation.

Selection criteria for inclusion in the study were: men over 18 years old; treated for a single recurrence within the prostatic bed after an initial RP and salvage or adjuvant EBRT; and approval of the treatment by the multidisciplinary uro-oncology team.

All men were initially treated with curative intent RP with lymph node sampling. In the case of BCR patients were treated with salvage or adjuvant radiotherapy with or without ADT. Radiation therapy was delivered by 3D conformal radiation planning or intensity-modulated radiotherapy (IMRT) to the prostatic bed with or without pelvic lymph node irradiation.

An isolated macroscopic relapse in the prostatic bed was confirmed with a combination of a [11 C] choline or prostate-specific membrane antigen (PSMA) positron emission tomography-computed tomography (PET/CT) to exclude any metastases and pelvic MRI with or without biopsies. All of the patients treated at our center and meeting the selection criteria are reported in this retrospective study.

### Planning and Treatment

**Figure 1:**
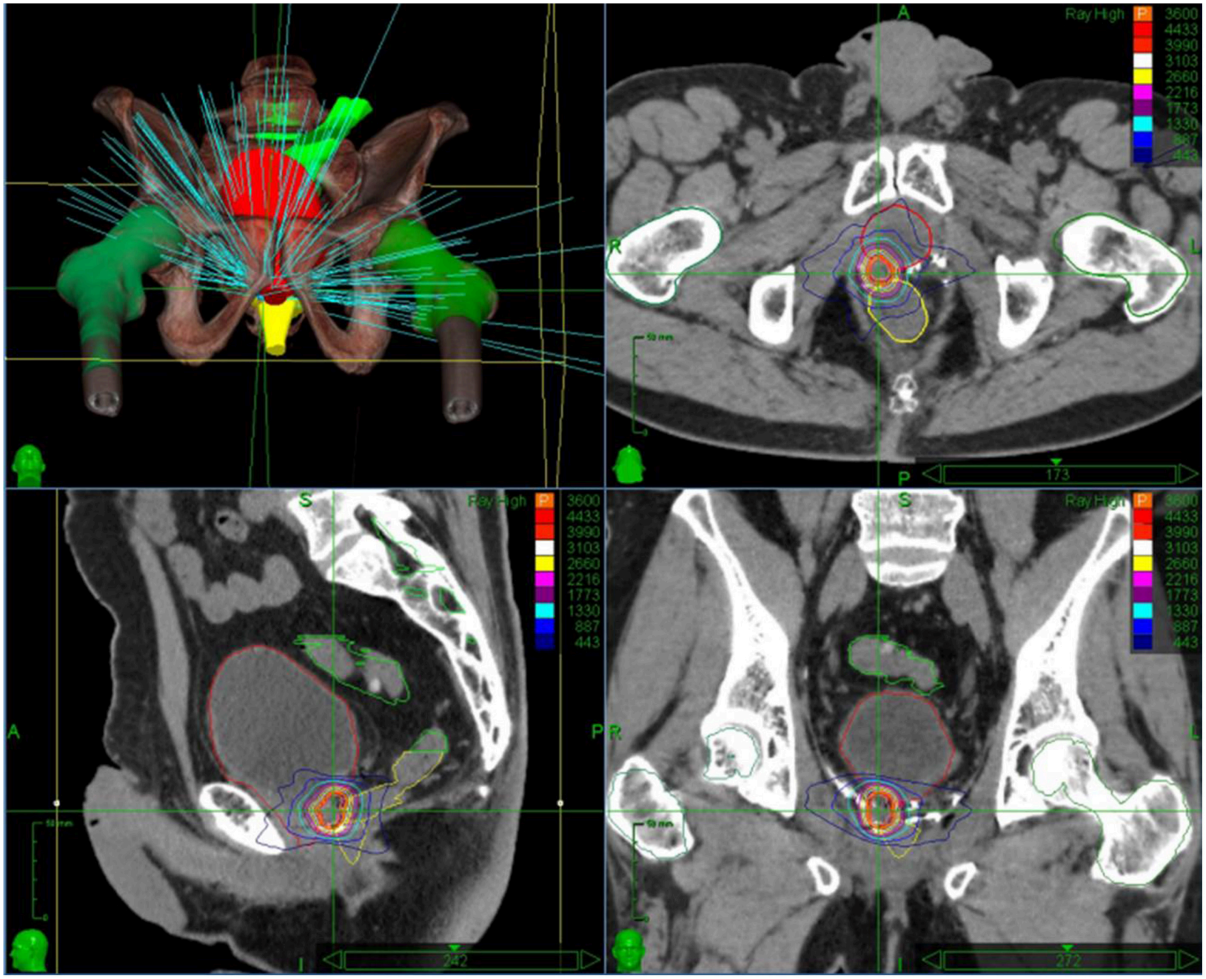
Stereotactic body radiation therapy treatment plan with dose distribution for an isolated macroscopic recurrence in the prostatic bed.

CyberKnife radiotherapy for macroscopic relapse in the prostatic bed was delivered for all patients. A total dose of 36 Gy was prescribed to the 80% isodose line (95% planning target volume, PTV, coverage) in 6 fractions of 6 Gy on alternating days (Figure 1). One internal gold fiducial was placed in contact with the lesion by an uro-radiologist with the help of MRI/ultrasound (US) fusion software. The fiducial was used for tracking the translational movements of the target lesion during SBRT. Delineation was made on a planning CT scan that was registered with the pre-treatment MRI (when the fiducial was visible on MRI) and the PET-CT to help with delineating the gross tumor volume (GTV). GTV was defined as macroscopic local recurrence on imaging. CTV corresponded to GTV. PTV was obtained from a 2 mm margin applied to CTV. Normal tissue constraints used for the planning were for the rectum V12 < 20%, V27 < 2cc, and for the bladder V12 < 15%,V27 < 5cc. These constraints were used for prostatic re-irradiation (16) and are actually used in a GETUG phase 2 protocol of prostatic re-irradiation ^1^. Priority was given to the respect of the normal tissue constraints used. PTV coverage was sacrificed if necessary.

### Follow-Up

After treatment, patients were seen by a radiation oncologist at 4 months and then every 6 months and were assessed using PSA level measurement and a clinical examination. Response to treatment was defined by a PSA divided by 2. Third BCR after SBRT was defined by 2 rises in PSA of ≥0.2 ng/mL above nadir (17). PET/CT was performed to assess the site of recurrence in cases of increasing PSA level.

Urinary, rectal, and sexual toxicity data were collected and scored according to the Common Terminology Criteria for Adverse Events (CTCAE) v4.0 questionnaire at baseline and at every follow-up consultation. Acute toxicity was defined as that occurring during the treatment and until 4 months after treatment.

### Statistics

Statistical analysis was undertaken by the Methodology and Biostatistics Unit (UMB) of the Oscar Lambret Cancer Centre and was performed using Stata software (StataCorp. 2013. Stata Statistical Software: Release 13. College Station, TX: StataCorp LP). Baseline characteristics were analyzed using descriptive methods. Categorical variables are presented as frequencies and percentages. Continuous variables are presented as medians with range and/or interquartile range, and means with standard deviation if justified. The numbers of missing data are specified for each variable.

## Results

Twelve patients with a median age of 58, were treated with SBRT between July 2011 and November 2017 for prostatic bed recurrence with a median follow-up of 34.2 months (range, 3.5–64.4 months). Initial treatment was RP with 50% pT2, 50% pT3, and 83% R1 margins and no patient had nodal invasion. Post-operative EBRT was performed in a median time of 6 months using 3D conformal radiation planning for 11 patients and IMRT for 1 patient, with a dose of 66 Gy for 11 patients and 72 Gy for 1 patient. Only 3/12 patients received ADT with EBRT for 6–12 months. The clinical characteristics before SBRT are summarized in Table 1.

**Table 1:** Patients' characteristics before stereotactic body radiation therapy.

**Patient**	**PSA pre-RP** **(ng/mL)**	**Gleason score at RP**	**pT**	**pN**	**pR**	**PSA pre-EBRT** **(ng/mL)**	**Time between RP and EBRT** **(months)**	**PSA nadir post-EBRT** **(ng/mL)**
1	17.3	3 + 4	pT2	pN0	R1	0.6	7	0.01
2	40	3 + 4	pT3a	pN0	R0	1.13	5	0.12
3	10.9	3 + 4	pT3b	pN0	R1	0.26	10	0.06
4	11	3 + 3	pT3a	pNx	R1	1.22	116	0.77
5	65	3 + 5	pT3b	pNx	R1		3	0.02
6	3.9	2 + 3	pT2	pN0	R1	0.22	18	0.04
7	4.6	3 + 4	pT2	pN0	R0	0.25	31	0.05
8	4.5	4 + 3	pT2	pN0	R1	1.49	6	0.03
9	10	3 + 4	pT3a	pN0	R1	0.47	25	0.06
10	20.3	4 + 3	pT3a	pN0	R1	1.02	4	0.3
11	10.5	3 + 4	pT2	pN0	R1	1.06	5	0.18
12	5.6	3 + 4	pT2	pN0	R1	0.24	1	0.01

*RP, radical prostatectomy; EBRT, external beam radiation therapy*.

Recurrence was determined using MRI for every patient and none of them had metastatic disease on PET scanning (all underwent choline PET-CT except 1 who underwent PSMA PET-CT). Local relapse biopsies were performed for 9 patients (75%), and recurrence was proven in 67% of them (6 patients). The median pre-SBRT PSA level was 1.13 ng/mL (0.57–5.71). SBRT was delivered in a median time of 77.6 months (range, 21. 4–160.8 months) after EBRT (Table 2). The treatment was delivered in 6 fractions over a median of 14 days. Two patients were treated with ADT concomitant with re irradiation for 6 months.

**Table 2:** Stereotactic body radiation therapy results.

**Patient**	**Time between EBRT and SBRT** **(months)**	**PSA** **pre-SBRT** **(ng/ml)**	**Use of ADT (time)**	**Volume PTV** **(cm^**3**^)**	**PSA** **at 4 months post-SBRT** **(ng/ml)**	**PSA** **nadir post-SBRT** **(ng/ml)**	**Recurrence**	**Recurrence** **free** **survival** **(months)**	**Recurrence localization**	**Recurrence localization** **(prostatic bed)**	**Treatment post-recurrence**
1	55	3	0	13.3	0.7	0.7	Yes	14	Prostatic bed	Outfield	ADT
2	71	1.8	0	6.3	0.2	0.1	Yes	26	Lymph nodes + metastases		ADT
3	108	5.7	0	6.4	7	NA	Yes	5	Prostatic bed + metastases	Outfield	ADT
4	21	2.1	6 months	4.1	0	0	Yes	22	Lymph nodes		ADT
5	34	0.8	0	4.3	0.6	0.3	Yes	42	Prostatic bed	Margin	ADT
6	125	0.7	0	1.2	0	0	No	–	–		–
7	50	0.6	0	2.1	0.6	0.2	No	–	–		–
8	76	1.2	6 months	12.8	0	0	No	–	–		–
9	123	0.9	0	2.9	0	0	No	–	–		–
10	79	1.1	0	4.5	1.8	1.8	Yes	4	Prostatic bed + Lymph nodes	Outfield	ADT
11	161	2	0	5.1	1	1	No	–	–		–
12	119	0.7	0	4.6	0.2	0.2	No	–	–		–

*EBRT, external beam radiation therapy; SBRT, stereotactic radiation therapy; ADT, androgen deprivation therapy; PTV, planning target volume*.

The median PTV was 4.5 cm^3^ (range, 1.2–13.3 cm^3^). The dosimetric data of the target volumes and the organs at risk are summarized in Table 3.

**Table 3:** Summary of the target volumes and the organs at risk dosimetric data.

	**CTV**	**PTV**		**Rectum**	**Bladder**
D98	35.9 (31.7–39.3)	34.6 (24.8–35.5)	V12 (%)	4.4 (0–10.0)	3.4 (0.8–13.8)
D95	36.8 (32.6–39.9)	35.9 (25.8–36.6)	V12 (cm^3^)	3.1 (0–14.8)	8.2 (1.4–21.0)
D50	41.0 (36.0–42.0)	39.4 (32.9–40.5)	V27 (%)	0.25 (0–1.1)	0.9 (0.1–3.8)
D2	42.2 (39.7–43.5)	42.1 (39.2–43.4)	V27 (cm^3^)	0.16 (0–1.7)	2.1 (0.2–5.6)

*Results are presented as median (minimum–maximum)*.

After treatment, response to treatment occurred in 10/12 patients (83%). The median nadir PSA was 0.16 ng/mL (0.01 to −0.97). A third BCR was observed for 6 patients (50%) after a median of 18 months (range, 4–42 months). The 1 and 2 years BCR-free survival rates were 79 and 56%, respectively. Eight patients (67%) were free of local recurrence. Four patients presented with distant relapse (lymph nodes or bone metastases). One patient died from non-PCa causes at 26 months.

The treatment was well-tolerated; Toxicity included 3 patients (25%) with grade 1 cystitis at 4 months, 1 patient (8.3%) with grade 2 proctitis at 4 months; 1 patient (12.5%) with grade 1 cystitis at 12 months and 2 patients with grade 2 urinary incontinence at 12 months, which was present before SBRT. No grade 3–4 toxicities were reported.

## Discussion

Currently, the standard of care for second BCR after RP and EBRT is long-term hormonal therapy, which is often not well-tolerated by patients (8). In cases of prostatic bed recurrence, we present one of the largest series of salvage SBRT after RP and EBRT. We show that this salvage treatment can be safe and could delay or avoid ADT.

The use of SBRT after PR and salvage or adjuvant EBRT has only previously been reported in 3 papers (Table 4). Janoray et al. described the results of 10 patients with a median follow-up of 11.7 months, Detti et al. described the results of 8 patients with a median follow-up of 10 months, and Zerini et al. described the results of 10 patients with a median follow up of 21.3 months (13–15).

**Table 4:** Summary of publications on salvage SBRT in prostatic bed after EBRT.

**Study**	**Number of patients (*n*=)**	**Median follow-up (months)**	**Biochemical response rate (%)**	**1 year BCR-free survival rate**	**Acute grade 1–2 urinary toxicity (%)**	**Acute grade 1–2 gastro-intestinal toxicity** **(%)**	**Acute grade 3–4 toxicity** **(%)**	**Late grade 1–2 urinary toxicity (%)**	**Late grade 1–2 gastro-intestinal toxicity** **(%)**	**Late grade 3–4 toxicity** **(%)**
Olivier	12	34.2	83	79	25	8	0	12.5	0	0
Janoray et al. (13)	10	11.7	90	80	14.3^*^	9.5^*^	0	0	0	0
Detti et al. (14)	8	10	88	NA	12.5	12.5	0	0	0	0
Zerini et al. (15)	10	21.3	NA	NA	10	10	0	10	10	0

*NA, No Available data*.

* *In this study, the toxicity results were presented for salvage SBRT both on prostate and prostatic bed*.

In our series, the biochemical response rate was 83%, compared to 90% in the study by Janoray et al. (13) and 88% in the Detti et al. (14) study. At 1 year, the BCR-free survival rate was 79% in our study compared to 80% in the study by Janoray et al. (13). We showed tumor control in 50% patients after a median follow-up of 34.2 months; this is comparable to the study by Zerini et al. (15) which showed that 40.6% of patients had no recurrence after a median follow-up of 21.3 months.

This treatment is well-tolerated with only 25% of patients experiencing grade 1 acute cystitis and 8% experiencing grade 2 acute proctitis at 4 months, and only 1 patient with grade 1 late cystitis at 1 year. Zerini et al. (15) reported that 10% of patients experienced grade 1 acute urinary toxicity, 10% experienced grade 2 acute rectal toxicity, 10% experienced grade 2 late urinary toxicity, and 10% experienced grade 1 late rectal toxicity. Detti et al. (14) reported 1 grade 2 acute genitourinary and gastrointestinal toxicity and no late toxicity, while Janoray et al. (13) reported 1 grade 2 acute genitourinary toxicity, and no grade ≥2 acute gastrointestinal or late toxicities.

The number of needed fiducials and their placement are still challenging questions (18). The use of US/MRI fusion is mandatory to place the gold marker in contact with the nodule and this procedure needs to be performed by a trained operator. The use of only 1 marker is theoretically less accurate for tracking as it does not take into account rotational movements. However, the placement of only one marker is easier and looks sufficient as rotation movement might be limited (19). Shakir et al. used 3 gold markers in the prostate bed for salvage therapy and showed that, despite the absence of the prostate, the implantation of gold markers was feasible; the motion of the fiducials was often limited to < 2 mm (20).

No biopsy was performed in the other series (12–14). Histological evidence of recurrence was obtained in 50% of our patients; 3 patients had not undergone biopsy and 3 presented with negative biopsy results. Histological evidence is important when considering a third local treatment. However, in cases of suspicious PSA kinetics associated with only local relapse on MRI and PET-CT, we suggest that neither the technical difficulty of performing a biopsy nor a negative biopsy should contraindicate the treatment after a decision by a multidisciplinary team.

The question of potentially lower radiosensitivity of the recurrence nodule after EBRT could justify the use of another modality of treatment for patients. Only few other local salvage treatments have been described after RP and EBRT. High intensity focal ultrasound (HIFU) has been described in very few studies as a salvage treatment after a second BCR (21). Murota-Kawano et al. (21) described the results of 4 patients with BCR after RP, of whom 3 received salvage EBRT; at 2 years, 2 of the 4 patients were biochemically disease-free and no treatment complication was reported. Salvage HIFU after RP and EBRT needs to be evaluated prospectively in a bigger cohort.

The main limitations of this study are the small number of patients included, the retrospective analyses of the data, the medium duration of follow-up, and the absence of a control arm. Among the 5 patients who presented with recurrence in the prostatic bed, only 1 presented with recurrence at the edge of the field, which shows good local efficacy. Among the 6 patients who relapsed, 2 (33%) presented with distant relapse immediately after treatment. This raises the question of appropriate selection of patients with only prostatic bed recurrence. The development of PSMA PET-CT might help the early detection of patients with metastatic disease and improve the selection of patients with only local relapse without any distant metastases in the future (22).

## Conclusion

This study shows that SBRT may be a promising treatment option for isolated macroscopic local recurrence after RP and EBRT, and could be considered a good alternative to long term ADT in this situation. However, these data are limited and need to be confirmed by a prospective study to validate the oncological and functional results of SBRT in this setting.

## Author Contributions

JO, LB, and DP contributed to the conception and design of the study. JO organized the database. JW performed the statistical analysis. JO and LB wrote the first draft of the manuscript. PP, JW, TL, and DP wrote sections of the manuscript. All authors contributed to manuscript revision, read and approved the submitted version.

### Conflict of Interest Statement

The authors declare that the research was conducted in the absence of any commercial or financial relationships that could be construed as a potential conflict of interest.

## Footnotes

^1^https://clinicaltrials.gov/ct2/show/NCT03438552
